# On a Derivative-Free Variant of King's Family with Memory

**DOI:** 10.1155/2015/514075

**Published:** 2015-03-25

**Authors:** M. Sharifi, S. Karimi Vanani, F. Khaksar Haghani, M. Arab, S. Shateyi

**Affiliations:** ^1^Department of Mathematics, Islamic Azad University, Shahrekord Branch, Shahrekord, Iran; ^2^Department of Mathematics and Applied Mathematics, University of Venda, Thohoyandou 0950, South Africa

## Abstract

The aim of this paper is to construct a method with memory according to King's family of methods without memory for nonlinear equations. It is proved that the proposed method possesses higher *R*-order of convergence using the same number of functional evaluations as King's family. Numerical experiments are given to illustrate the performance of the constructed scheme.

## 1. Introduction

Many problems arising in diverse disciplines of mathematical sciences can be described by a nonlinear equation of the following form (see, e.g., [1]):

(1) $$\begin{array}{c} f\left(x\right)=0, \end{array}$$

where *f* : *D*⊆ℝ → ℝ is a sufficiently differentiable function in a neighborhood *D* of a simple zero *α* of (1). If we are interested in approximating the root *α*, we can do it by means of an iterative fixed-point method in the following form:

(2) $$\begin{array}{c} x_{k+1}=\psi \left(x_{k}\right),\;k\geq 0, \end{array}$$

provided that the starting point *x*_0_ is given.

In this work, we are concerned with the fixed-point methods that generate sequences presumably convergent to the true solution of a given single smooth equation. These schemes can be divided into one-point and multipoint schemes. We remark that the one-point methods can possess high order by using higher derivatives of the function, which is expensive from a computational point of view. On the other hand, the multipoint methods are allowing the user not to waste information that had already been used. This approach provides the construction of efficient iterative root-finding methods [2].

In such circumstance, special attention is devoted to multipoint methods with memory that use already computed information to considerably increase convergence rate without additional computational costs. This would be the focus of this paper.

Traub in [2] proposed the following method with memory (TM):

(3) $$\begin{array}{c} w_{k}=x_{k}+\beta_{k}f\left(x_{k}\right),\;\beta_{k}=-\frac{1}{f\left[x_{k},x_{k-1}\right]},\;k=0,1,2,\ldots ,\\ x_{k+1}=x_{k}-\frac{f\left(x_{k}\right)}{f\left[x_{k},w_{k}\right]}, \end{array}$$

with the order of convergence $1+\sqrt{2}$.

The iterative methods with memory can improve the order of convergence of the without memory method without any additional functional calculations, and this results in a higher computational efficiency index. We remark that it is assumed that an initial approximation *x*_0_ close enough to the sought simple zero and *β*_0_ are given for iterative methods of type (3).

Recently, authors in [3] designed an approach to make derivative-free families with low complexity out of optimal methods. In fact, they conjectured that every time that one applies the approximation of the derivative *f*′(*x*_*n*_) ≈ *f*[*x*_*n*_, *w*_*n*_], with *w*_*n*_ = *x*_*n*_ + *βf*(*x*_*n*_)^*l*^, on an optimal order 2*q*, we will need *l* ≥ *q* for preserving the order of convergence.

For instance, choosing the well-known optimal two-step family of King (KM) [4],

(4) $$\begin{array}{c} y_{k}=x_{k}-\frac{f\left(x_{k}\right)}{f^{\prime }\left(x_{k}\right)},\;k=0,1,2,\ldots ,\\ x_{k+1}=y_{k}-\frac{f\left(y_{k}\right)}{f^{\prime }\left(x_{k}\right)}\frac{f\left(x_{k}\right)+\gamma f\left(y_{k}\right)}{f\left(x_{k}\right)+\left(\gamma -2\right)f\left(y_{k}\right)},\;\gamma \in R, \end{array}$$

and the conjecture of Cordero-Torregrosa, one may propose the following method (DKM):

(5) $$\begin{array}{c} y_{k}=x_{k}-\frac{f\left(x_{k}\right)}{FD},\;k=0,1,2,\ldots ,\\ x_{k+1}=y_{k}-\frac{f\left(y_{k}\right)}{FD}\frac{f\left(x_{k}\right)+\gamma f\left(y_{k}\right)}{f\left(x_{k}\right)+\left(\gamma -2\right)f\left(y_{k}\right)},\;\gamma \in R, \end{array}$$

wherein

(6) FD=f(xk)−f(wk)xk−wk, wk=xk+βfxk2,β∈R∖0.

In this work, we propose a two-step method with memory possessing a high efficiency index according to the well-known family of King's methods (5).

Our inspiration and motivation for constructing a higher-order method are linked in a direct manner with the fundamental concept of numerical analysis that any numerical method should give as accurate as possible output results with minimal computational cost. To state the matter differently, it is necessary to pursue methods of higher computational efficiency.

For more background concerning this topic, one may refer to [5, 6].

The paper is organized as follows. In Section 2, the aim of this paper is presented by contributing an iterative method with memory based on (5) for solving nonlinear equations. The proposed scheme is an extension over (4) and has a simple structure with an increased computational efficiency. In Section 3, we compare the theoretical results by applying the definition of efficiency index and further supports are furnished whereas numerical reports are stated. Some concluding remarks will be drawn in Section 4 to end the paper.

## 2. A New Method with Memory

In this section, we propose the following iterative method with memory based on (5):

(7) $$\begin{array}{c} y_{k}=x_{k}-\frac{f\left(x_{k}\right)}{FD},\;w_{k}=x_{k}+\beta_{k}f{\left(x_{k}\right)}^{2},\;k=0,1,2,\ldots ,\\ x_{k+1}=y_{k}-\frac{f\left(y_{k}\right)}{FD}\frac{f\left(x_{k}\right)+\gamma f\left(y_{k}\right)}{f\left(x_{k}\right)+\left(\gamma -2\right)f\left(y_{k}\right)},\;\gamma =-\frac{1}{2}, \end{array}$$

wherein the self-accelerating parameter is *β*_*k*_. The error equation of (5) is (*γ* = −1/2)

(8) $$\begin{array}{c} e_{k+1}=-c_{2}\left(f^{\prime }{\left(\alpha \right)}^{2}\beta c_{2}+c_{3}\right)e_{k}^{4}+O\left(e_{k}^{5}\right), \end{array}$$

where *c*_*j*_ = (1/*j*!)(*f* ^(*j*)^(*α*)/*f*′(*α*)). We now must find a way so as to vanish the asymptotic error constant *η* = −*c*_2_(*f*′(*α*)^2^ *βc*_2_ + *c*_3_).

Toward this goal, one can increase the *R*-order by considering the following substitution:

(9) $$\begin{array}{c} \beta =-\frac{c_{3}}{f^{\prime }{\left(\alpha \right)}^{2}c_{2}}. \end{array}$$

Since the zero is not known, relation (9) cannot be used in its exact form and we must approximate it recursively. This builds a variant with memory for King's family by using

(10) $$\begin{array}{c} \beta_{k}\approx -\frac{{\overset{-}{c}}_{3}}{{\overset{-}{f}}^{\prime }{\left(\alpha \right)}^{2}{\overset{-}{c}}_{2}}, \end{array}$$

where ${\overset{-}{c}}_{j}\approx c_{j}$. Now if we consider *N*_3_(*t*) to be Newton's interpolation polynomial of third degree set through four available approximations *x*_*k*_, *x*_*k*−1_, *y*_*k*−1_, *w*_*k*−1_ at the end of each cycle, we can propose the following new method with memory:

(11) βk=−N3′′′xk3N3′xk2N3′′(xk),yk=xk−fxkFD, wk=xk+βkfxk2,k=0,1,2,…,xk+1=yk−f(yk)FDf(xk)−1/2f(yk)f(xk)−5/2f(yk).

Note that, for example, we have the following formulation for the interpolating polynomial:

(12) N3′xk=ddtN3(t)t=xk=f[xk,xk−1]+f[xk,xk−1,yk−1](xk−xk−1) +f[xk,xk−1,yk−1,wk−1](xk−xk−1)(xk−yk−1).

Acceleration in convergence for (11) is based on the use of a variation of one free nonzero parameter in each iterative step. This parameter is calculated using information from the current and previous iteration(s) so that the developed method may be regarded as method with memory according to Traub's classification [2].

We are at the time to write about the theoretical aspects of our proposed solver (11).

**Theorem 1 .**

Let the function *f*(*x*) be sufficiently differentiable in a neighborhood of its simple zero *α*. If an initial approximation *x*_0_ is sufficiently close to *α*, then, the *R*-order of convergence of the two-step method (11) with memory is at least 4.23607.

**Proof**

Let {*x*_*k*_} be a sequence of approximations generated by an iterative method. The error relations with the self-accelerating parameter *β* = *β*_*k*_ for (11) are in what follows:

(13) $$\begin{array}{c} {\hat{e}}_{k}=w_{k}-\alpha \sim c_{k,1}e_{k}, \end{array}$$

(14) $$\begin{array}{c} {\tilde{e}}_{k}=y_{k}-\alpha \sim c_{k,2}e_{k}^{2}, \end{array}$$

(15) $$\begin{array}{c} e_{k+1}=x_{k+1}-\alpha \sim c_{k,4}e_{k}^{4}. \end{array}$$

Using a symbolic computations, we attain that

(16) $$\begin{array}{c} -c_{2}\left(f^{\prime }{\left(\alpha \right)}^{2}\beta c_{2}+c_{3}\right)\sim e_{k-1}. \end{array}$$

Substituting the value of −*c*_2_(*f*′(*α*)^2^ *βc*_2_ + *c*_3_) from (16) in (15), one may obtain

(17) $$\begin{array}{c} e_{k+1}\sim c_{k,4}e_{k-1}e_{k}^{4}. \end{array}$$

Note that in general we know that the error equation should read *e*_*k*+1_ ~ *Ae*_*k*_^*p*^, where *A* and *p* are to be determined. Hence, one has *e*_*k*_ ~ *Ae*_*k*−1_^*p*^, and subsequently

(18) $$\begin{array}{c} e_{k-1}\sim A^{-1/p}e_{k}^{1/p}. \end{array}$$

Thus, it is easy to obtain

(19) $$\begin{array}{c} e_{k}^{p}\sim A^{-1/p}Ce_{k}^{4+1/p}, \end{array}$$

wherein *C* is a constant. This results in

(20) $$\begin{array}{c} p=4+\frac{1}{p}, \end{array}$$

with two solutions {−0.236068,4.23607}. Clearly the value for *p* = 4.23607 is acceptable and would be the convergence *R*-order of method (11) with memory. The proof is complete.

The increase of *R*-order is attained without any (new) additional function calculations so that the novel method with memory possesses a high computational efficiency index. This technique is an extension over scheme (5) to increase the *R*-order from 4 to 4.23607.

The accelerating method (11) is new, simple, and useful, providing considerable improvement of convergence rate without any additional function evaluations in contrast to the optimal two-step methods without memory.

We also remark that an alternative form of our proposed method with memory could be deduced using backward finite difference formula at the beginning of the first substep and a minor modification in the accelerators; that is to say, we have the following alternative method with memory possessing 4.23607 as its *R*-order (APM) as well:

(21) $$\begin{array}{c} \beta_{k}=\frac{N_{3}^{\prime \prime \prime }\left(x_{k}\right)}{3N_{3}^{\prime }{\left(x_{k}\right)}^{2}N_{3}^{\prime \prime }\left(x_{k}\right)},\\ y_{k}=x_{k}-\frac{f\left(x_{k}\right)}{FD},\;\;w_{k}=x_{k}-\beta_{k}f{\left(x_{k}\right)}^{2},\\ x_{k+1}=y_{k}-\frac{f\left(y_{k}\right)}{FD}\frac{f\left(x_{k}\right)-1/2f\left(y_{k}\right)}{f\left(x_{k}\right)-5/2f\left(y_{k}\right)}. \end{array}$$

**Theorem 2 .**

Let the function *f*(*x*) be sufficiently differentiable in a neighborhood of its simple zero *α*. If an initial approximation *x*_0_ is sufficiently close to *α*, then, the *R*-order of convergence of the two-step method (21) with memory is at least 4.23607.

**Proof**

The proof of this theorem is similar to Theorem 1. It is hence omitted.

## 3. Numerical Computations

Computational efficiency of different iterative methods with and without memory can be measured in a prosperous manner by applying the definition of efficiency index. For an iterative method with convergence (*R*-)order *r* that requires *θ* functional evaluations, the efficiency index (also named computational efficiency) is calculated by Ostrowski-Traub's formula [2]:

(22) $$\begin{array}{c} E=r^{1/\theta }. \end{array}$$

According to this, we find

(23) ESM≈1.4142<E(3)≈1.5737=E(4)≈1.5874=E(5)≈1.5874<E(11)≈1.6180,

where SM is the quadratically convergent method of Steffensen without memory [7].

It should be remarked that Džunić in [8] designed an efficient one-step Steffensen-type method with memory possessing $\left(1/2)(3+\sqrt{17}\right)$ *R*-order of convergence as follows:

(24) $$\begin{array}{c} w_{k}=x_{k}+\beta_{k}f\left(x_{k}\right),\\ \beta_{k}=-\frac{1}{N_{2}^{\prime }\left(x_{k}\right)},\;\;p_{k}=-\frac{N_{3}^{\prime \prime }\left(w_{k}\right)}{2N_{3}^{\prime }\left(w_{k}\right)},\\ x_{k+1}=x_{k}-\frac{f\left(x_{k}\right)}{f\left[x_{k},w_{k}\right]+p_{k}f\left(w_{k}\right)}, \end{array}$$

and Cordero et al. in [9] presented a two-step biparametric Steffensen-type iterative method with memory possessing seventh *R*-order of convergence:

(25) $$\begin{array}{c} w_{k}=x_{k}+\beta_{k}f\left(x_{k}\right),\;\;\beta_{k}=-\frac{1}{N_{3}^{\prime }\left(x_{k}\right)},\\ p_{k}=-\frac{N_{4}^{\prime \prime }\left(w_{k}\right)}{2N_{4}^{\prime }\left(w_{k}\right)},\\ y_{k}=x_{k}-\frac{f\left(x_{k}\right)}{f\left[x_{k},w_{k}\right]+p_{k}f\left(w_{k}\right)},\\ x_{k+1}=y_{k}-\frac{f\left(y_{k}\right)}{f\left[x_{k},y_{k}\right]+f\left[w_{k},x_{k},y_{k}\right]\left(y_{k}-x_{k}\right)}. \end{array}$$

Note that our main aim was to develop King's family in terms of efficiencies index and was not to achieve the highest possible efficiency index.

Although these methods possess higher computational efficiency indices than our proposed method (11), we exclude them from numerical comparisons since our method is not a Steffensen-type method and it is a Newton-type method with memory. For more refer to [10].

Now, we apply and compare the behavior of different methods for finding the simple zeros of some different nonlinear test functions in the programming package Mathematica [11] using multiple precision arithmetic to clearly reveal the high *R*-order of PM and APM. We compare methods with the same number of functional evaluations per cycle.

We notice that, by applying any root solver with local convergence, a special attention must be paid to the choice of initial approximations. If initial values are sufficiently close to the sought roots, then the expected (theoretical) convergence speed is obtainable in practice; otherwise, the iterative methods show slower convergence, especially at the beginning of the iterative process.

In this section, the computational order of convergence (coc) has been computed by

(26) $$\begin{array}{c} \text{coc}=\frac{\ln\left|f\left(x_{k}\right)/f\left(x_{k-1}\right)\right|}{\ln\left|f\left(x_{k-1}\right)/f\left(x_{k-2}\right)\right|}. \end{array}$$

The calculated value coc estimates the theoretical order of convergence well when pathological behavior of the iterative method (i.e., slow convergence at the beginning of the implemented iterative method, oscillating behavior of approximations, etc.) does not exist.

Here the results of comparisons for the test functions are given by applying 1000 fixed floating point arithmetic using the stop termination |*f*(*x*_*k*_)|≤10^−100^.

**Example 3 .**

We consider the following nonlinear test function in the interval *D* = [1.5,2.5]:

(27) $$\begin{array}{c} f\left(x\right)=\left(x-2\tan\left(x\right)\right)\left(x^{3}-8\right), \end{array}$$

using the initial approximation *x*_0_ = 1.7. The results are provided in Table 1.

In this section, we have used *β*_0_ = 0.0001 whenever required. Furthermore, for DKM we considered *γ* = −1/2.

**Example 4 .**

We compare the behavior of different methods for finding the complex solution of the following nonlinear equation:

(28) $$\begin{array}{c} g\left(x\right)=\left(-1+2I\right)+\frac{1}{x}+x+\sin\left(x\right), \end{array}$$

using the initial approximation *x*_0_ = 1 − 3*I* where *α* = 0.28860 ⋯ −1.24220 ⋯ *I*. The results for this test are given in Table 2.

It is evident from Tables 1 and 2 that approximations to the roots possess great accuracy when the proposed method with memory is applied. Results of the fourth iterate in Tables 1 and 2 are given only for demonstration of convergence speed of the tested methods and in most cases they are not required for practical problems at present.

We also incorporated and applied the developed methods with memory (11) and (21) for different test examples and obtained results with the same behavior as above. Hence, we could mention that the theoretical results are upheld by numerical experiments and thus the new method is good with a high computational efficiency index.

## 4. Summary

In this paper, we have proposed a new two-step Steffensen-type iterative method with memory for solving nonlinear scalar equations. Using one self-correcting parameter calculated by Newton interpolatory polynomial, the *R*-order of convergence of the constructed method was increased from 4 to 4.23607 without any additional calculations.

The new method was compared in performance and computational efficiency with some existing methods by numerical examples. We have observed that the computational efficiency index of the presented method with memory is better than those of other existing two-step King-type methods.

## Figures and Tables

**Table 1:** Results of comparisons for Example 3 and to find α = 2.

Methods	|*f*(*x*_1_)|	|*f*(*x*_2_)|	|*f*(*x*_3_)|	|*f*(*x*_4_)|	coc
KM	18.577	64890.	7.2226 × 10^10^	3.2493 × 10^9^	—
OM	4.7484	0.0023129	1.3928 × 10^−16^	1.8313 × 10^−69^	4.00000
DKM	0.53362	5.3207 × 10^−7^	5.2711 × 10^−31^	5.0774 × 10^−127^	4.00000
PM	0.53362	1.9202 × 10^−6^	3.6106 × 10^−30^	1.6392 × 10^−130^	4.22928

**Table 2:** Results of comparisons for Example 4.

Methods	|*f*(*x*_1_)|	|*f*(*x*_2_)|	|*f*(*x*_3_)|	|*f*(*x*_4_)|	coc
KM	2.0873	0.0095650	7.7971 × 10^−12^	3.4597 × 10^−48^	4.00000
OM	0.81344	0.0010884	1.5476 × 10^−15^	6.3280 × 10^−63^	4.00000
DKM	2.1909	0.013379	2.9909 × 10^−11^	7.5008 × 10^−46^	4.00000
PM	2.1909	0.0011772	7.0556 × 10^−16^	8.4197 × 10^−68^	4.23539
APM	1.9861	0.00089226	2.3251 × 10^−16^	7.5243 × 10^−70^	4.23526
